# Laryngeal tuberculosis mimicking malignancy: A case report

**DOI:** 10.1002/ccr3.2882

**Published:** 2020-04-21

**Authors:** Mohamed Eltilib, William Boyd, Israel Saramago, Frederic Askin, Carlos Zamora

**Affiliations:** ^1^ University of North Carolina School of Medicine Chapel Hill North Carolina; ^2^ Department of Radiology University of North Carolina School of Medicine Chapel Hill North Carolina; ^3^ Department of Pathology University of North Carolina School of Medicine Chapel Hill North Carolina

**Keywords:** case report, granulomatous disease, larynx, miliary, tuberculosis

## Abstract

Laryngeal involvement in tuberculosis is rare and may mimic malignancy. We report the case of a 26‐year‐old female who presented with sore throat for several months. CT revealed an infiltrative laryngeal mass and upper lobe pulmonary opacities. Laryngoscopic biopsy confirmed necrotizing granulomatous inflammation with positive culture for *Mycobacterium tuberculosis*.

## Graphical abstract

Laryngeal involvement is a rare manifestation of tuberculosis and may be misdiagnosed as a primary malignancy in nonendemic areas. A high degree of clinical suspicion is warranted in patients with an appropriate travel history to endemic areas.

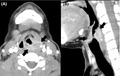

## 1 INTRODUCTION

Laryngeal tuberculosis is rare, and its diagnosis requires a high degree of clinical suspicion. Involvement of the larynx is seen in <1% of patients with tuberculosis and can present with insidious manifestations including persistent laryngitis.^1^, ^2^ The pathologic hallmark of tuberculosis is formation of caseating granulomas which can give rise to an infiltrative mass that may mimic malignancy.^2^, ^3^ Notably, laryngeal tuberculosis can develop with or without the presence of pulmonary disease. History of travel to endemic areas is important and identification of such patients is crucial to ensure that biopsy and other procedures are performed under safe conditions (ie, airborne precautions, negative pressure ventilation, N95 respirator use, and others). The aim of this report is to present a case of laryngeal tuberculosis in a patient who presented with an infiltrative laryngeal mass and to provide clues that may facilitate a prompt diagnosis.

## 2 CASE REPORT

**Figure 1:**
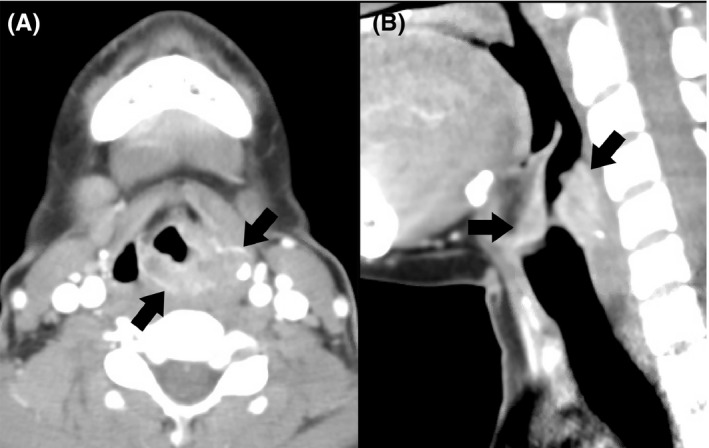
Axial (A) and sagittal (B) contrast‐enhanced axial CT images demonstrate an infiltrative, heterogeneously enhancing mass centered in the supraglottis involving both aryepiglottic folds with obliteration of the left piriform sinus (arrows). There is partial infiltration of the paraglottic fat

**Figure 2:**
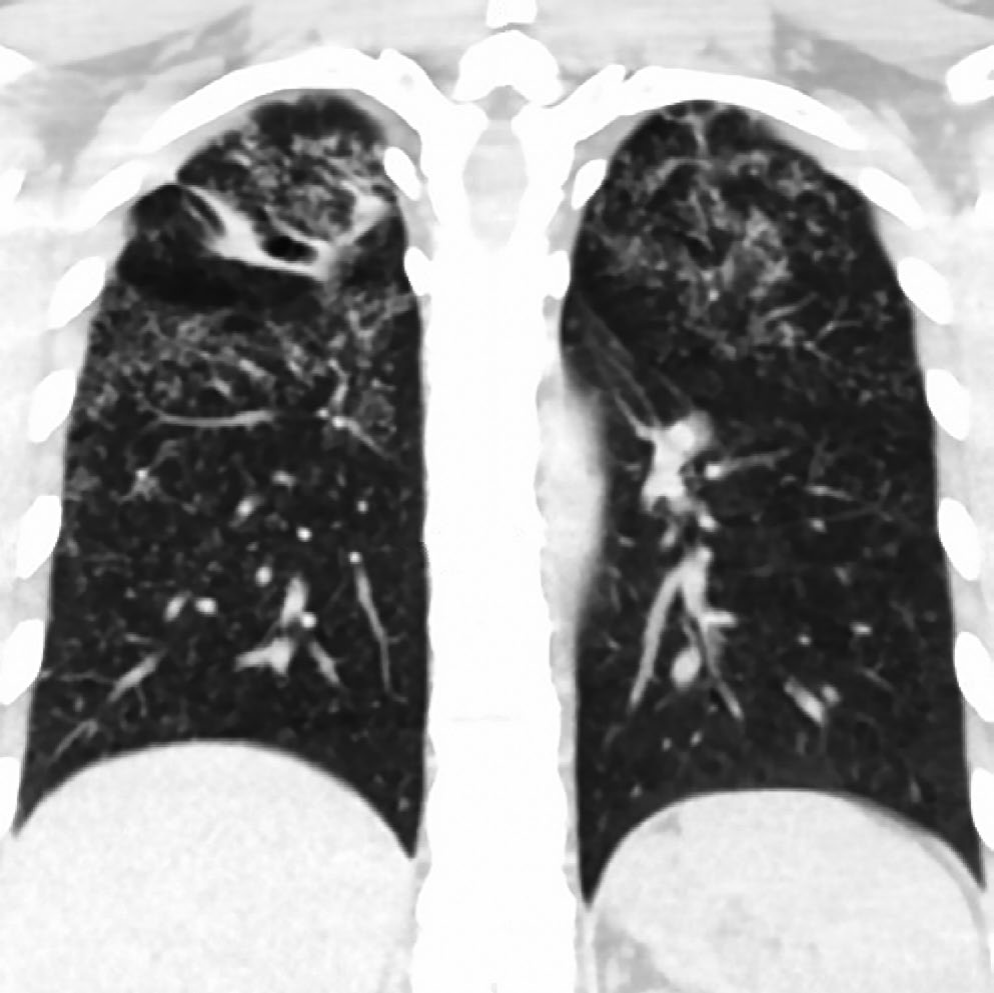
Coronal noncontrast CT image of the chest shows upper lobe predominant infiltrates with a miliary pattern and bronchiectasis positive for mycobacterium tuberculosis in sputum

**Figure 3:**
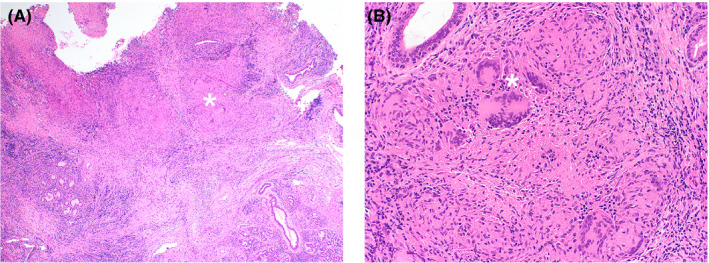
Low power view (A) of excisional biopsy showing numerous necrotizing granulomas (*) in the submucosa. Higher power (B) image showing necrotizing granulomatous inflammation with giant cells (*)

A 26‐year‐old female without significant past medical history presented to the emergency room complaining of sore throat, cough, and hoarseness for over 3 months. She had tried over the counter medications for the common cold and had received antibiotics at an outside clinic for a presumed diagnosis of bacterial pharyngitis, without symptom improvement. There was no history of fevers, night sweats, weight loss, or hemoptysis. Physical examination was unremarkable. Social and occupational history were significant for growing up in Mexico and having been a plastic fiber optic worker. Initial laboratory examinations revealed a normal complete blood count and complete metabolic panel. Contrast‐enhanced CT of the neck revealed an infiltrative supraglottic mass with partial glottic extension (Figure 1) and multiple nodular pulmonary opacities in the upper lobes of the lungs (Figure 2). The patient's age, social history, and presence of upper lobe opacities raised concern for tuberculosis as a possible etiology, and she was subsequently placed on airborne isolation. Acid‐fast bacilli (AFB Ziehl‐Neelsen) and Fite stains on sputum were negative; however, cultures yielded *Mycobacterium tuberculosis.* Other relevant laboratory workup included positive ANA, positive ANCA cytoplasmic pattern, negative HIV, and positive interferon‐gamma release assay. Flexible laryngoscopy demonstrated a polypoid lesion largely involving the aryepiglottic folds and arytenoid cartilage with extension to the interarytenoid space, false and true vocal folds, and hypopharynx. An excisional biopsy was then performed which revealed numerous areas of necrotizing granulomatous inflammation (Figure 3). The patient was started on rifampin, isoniazid, pyrazinamide, ethambutol, and vitamin B6 (ie, RIPE + B6) and was discharged 7 days after admission under this regimen to be followed up at her community hospital. Her respective health department was contacted for screening and treatment of close contacts.

## 3 DISCUSSION

Laryngeal tuberculosis is a rare condition that requires a high degree of clinical suspicion for diagnosis. Laryngeal involvement develops most commonly secondary to bronchogenic, hematogenous, or lymphatic spread from advanced pulmonary disease. Primary laryngeal tuberculosis without pulmonary disease is much less common and is presumed to arise from direct invasion of the larynx via inhalation.^2^ The most common sites of laryngeal involvement are the epiglottis, true vocal cords, and false vocal cords, although the disease can be trans‐spatial and affect any tissue.

In order to avoid a delay in diagnosis and management, the possibility of tuberculosis should always be entertained in patients who have traveled to endemic areas and who present with chronic hoarseness, odynophagia, and/or weight loss. In nonendemic regions, laryngeal tuberculosis may be easily misdiagnosed as primary malignancy given its mass‐like and infiltrative appearance as well as its relatively nonspecific presentation. However, laryngeal cancer is typically seen in older individuals and is notably an unusual diagnosis before age 40. Other granulomatous processes that can present with laryngeal involvement include granulomatosis with polyangiitis, sarcoidosis, and syphilis.^3^

In cases of suspected laryngeal tuberculosis, chest radiography or chest CT are appropriate first steps to evaluate for pulmonary involvement. In a case series that analyzed 127 cases of laryngeal tuberculosis in the United States between 1970 and 2012, radiographic findings of pulmonary tuberculosis were reported in approximately 86% of cases.^4^ In that case series, the most common presenting symptoms were hoarseness, cough, and dysphagia. Most patients with laryngeal tuberculosis will also have pulmonary involvement, and therefore, chest radiography or CT are appropriate initial evaluations. Other imaging tests for extrapulmonary tuberculosis may be performed and are based on specific clinical findings. These usually include CT for abdominal or pelvic disease and MRI with and without intravenous contrast when there is suspected involvement of the spine or central nervous system. Positron emission tomography (PET)/CT has the potential to delineate the extent of extrapulmonary disease due to avid uptake of F‐fluoro‐2‐deoxyglucose (FDG) by the tuberculous lesions. ^5^ However, PET/CT does not currently play a widely accepted role in screening for extrapulmonary disease, probably in part due to cost and availability. Sputum cultures and AFB smears should also be obtained in all patients with suspected laryngeal tuberculosis. Direct laryngoscopy following appropriate infectious precautions can confirm the diagnosis and exclude malignancy or other chronic granulomatous processes. However, some authors advocate performing biopsy in patients with cervical lymphadenopathy, risk factors for laryngeal cancer, or in those who do not respond to anti‐tuberculous therapy.^6^

The Infectious Diseases Society of America (ISDA) clinical practice guidelines for the treatment of tuberculosis state that the ultimate objectives are eradication of *Mycobacterium tuberculosis*, prevention of relapse, and prevention of transmission to others in the community. First‐line regimen for adults with drug‐susceptible tuberculosis consists of an intensive phase of 2 months of isoniazid, rifampin, pyrazinamide, and ethambutol followed by a continuation phase of 4 months of isoniazid and rifampin.^7^ In a case series of 127 patients with laryngeal tuberculosis, a mortality rate of 3% (4/127) was observed with a standard antituberculosis regimen and a duration of treatment ranging from 6 to 12 months.^4^ Another study examining 5 patients with confirmed laryngeal tuberculosis found that the larynx returned to its normal appearance after an average of 18 weeks of treatment with anti‐tuberculous drugs.^8^ Rarely, fibrosis of laryngeal tissues can occur as the larynx heals, resulting in chronic dysphonia, glottic stenosis, or subglottic stenosis. Surgical treatment may be warranted if laryngeal fibrosis occurs. However, this has been infrequently reported in the literature and is not a common complication.^9^ Current ISDA recommendations also state that sputum should be obtained during treatment for AFB smear and culture once a month until two consecutive cultures are negative.^7^

## 4 CONCLUSION

Laryngeal involvement is a rare manifestation of tuberculosis and may mimic primary malignancy. A high level of clinical suspicion is warranted for safe laryngeal biopsy. The possibility of this diagnosis should be considered particularly in patients with the appropriate travel history to endemic areas.

## CONFLICT OF INTEREST

None.

## AUTHOR CONTRIBUTIONS

ME performed a literature search and drafted the manuscript. WB provided revisions and drafted portions of the manuscript. IS drafted portions of the manuscript, provided revisions, and acquired the images for Figures 1 and 2. FA provided the pathology slides with captions and revised the manuscript for intellectual content. CZ conceived the project, was the designated faculty advisor, revised the manuscript for intellectual content, and approved the final draft.
